# Longitudinal assessment of the relationship between frailty and social relationships among Japanese older adults: a random intercept cross-lagged panel model

**DOI:** 10.1186/s12889-024-18234-1

**Published:** 2024-03-05

**Authors:** Mingyu Cui, Dandan Jiao, Yang Liu, Yantong Zhu, Xiang Li, Zhu Zhu, Jinrui Zhang, Afsari Banu Alpona, Yanlin Wang, Meiling Qian, Yuko Sawada, Kumi Watanabe Miura, Taeko Watanabe, Emiko Tanaka, Tokie Anme

**Affiliations:** 1 School of Comprehensive Human Science, University of Tsukuba, Tsukuba, Japan; 2 Department of Physical Therapy, Morinomiya University of Medical Sciences, Osaka, Japan; 3 RIKEN Center for Advanced Intelligence Project, Tokyo, Japan; 4 Faculty of Nursing, Shukutoku University, Chiba, Japan; 5 Faculty of Nursing, Musashino University, Tokyo, Japan; 6 Faculty of Medicine, University of Tsukuba, Tsukuba, Japan; 7 Department of Nursing, The First Affiliated Hospital, and College of Clinical Medicine, Henan University of Science and Technology, Luoyang, China

**Keywords:** Older people, Random intercept cross-lagged panel model, Frailty, Social relationships, Longitudinal study

## Abstract

**Objectives:**

This study aimed to explore the bidirectional association between frailty and social relationships in older adults while distinguishing between interpersonal and intrapersonal effects.

**Methods:**

A prospective cohort study of community-dwelling older adults was conducted in Japan in three waves spanning six years with follow-ups in every three years. Random intercept cross-lagged panel model was used to explore temporal associations between frailty and social relationships.

**Results:**

Data for 520 participants (mean age 73.02 [SD 6.38] years, 56.7% women) were analyzed. Across individuals, frailty was associated with social relationships (β = -0.514, *p* < 0.001). At the interpersonal level, frailty was cross-sectionally associated with social relationships separately at T1(β = -0.389, *p* < 0.01), T2 (β = -0.343, *p* < 0.001) and T3 (β = -0.273, *p* < 0.05). Moreover, social relationships were associated with subsequent increases in symptoms of frailty in all measurement waves (β = -0.332, *p* < 0.001; β = -0.169, *p* < 0.01) and vice versa (β = -0.149, *p* < 0.05; β = -0.292, *p* < 0.001).

**Conclusions:**

The results suggest that frailty was associated with lower levels of social relationships. Frailty improvement programs can be combined with interventions to enhance social relationships, which will be beneficial in preventing frailty. The results emphasize the importance of combining clinical treatments of frailty with interventions to improve social relationships.

## Introduction

Global aging is increasing, and frailty is putting more pressure on health care systems. Frailty in older adults is a common condition that has been gaining attention in recent years, affecting more than 25% of people over the age of 85 [1]. Frailty is an evolving concept, normally describes an altered health status following a stressful event, that leaves one vulnerable to the effects of poorly balanced resolutions in the body [2, 3], and frequently leads to negative health consequences such as decreased functional status, hospitalization, disability, and death [2, 4, 5]. Research have shown that frailty is reversible and can be improved by interventions [2, 6]. Therefore, prevention of frailty in older people is an important issue in healthy aging.

Previous studies have established an association between frailty and a number of factors, including older age [7], poor economic status [8], low educational attainment [9], depression [10] and schizophrenia [11].

In addition to these, social factors, e.g., relationships, are also important determinants of frailty. Social relationships are the associations between individuals and their social environment [12] and have a direct impact on health outcomes regardless of the individual’s state of stress [13]. In recent years, a growing number of studies have begun to explore the connection between social relationships and frailty using population-based samples, however, the direction and mechanism of this connection is not fully understood [14]. For example, poor social relationships may be a risk factor for frailty; conversely, deterioration in social relationships may also lead to deterioration in health status, thereby exacerbating symptoms of frailty. It is perhaps even more likely that there is a vicious cycle in which deteriorating frailty weakens social relationships and health, which in turn exacerbates symptoms of frailty. Regardless of the cause-and-effect relationship, clarifying this relationship is critical to the clinical management of frailty patients as well as to interventions.

Previous studies revealed that social relationships predicted subsequent frailty. In a study of older Korean, frailty is more likely to occur among people who have less contact with others, as the frequency of contact with friends is most associated with frailty [15]. Similarly, in a one-year follow-up of community-dwelling older adults in China, the enrichment of social relationships positively influenced frailty by affecting the incidence of depression and physical activity [16]. However, few studies have focused on associations in the opposite direction, i.e., impact of frailty on social relationships. A small number of relevant studies exist but produce varied results. A longitudinal study in Japan showed that in the subfield of frailty, lower stepping speed and body strength were essential risk factors for subsequent declines in social relationships [17]. Whereas, a study in Amsterdam showed that the small social network of frail older adults existed at baseline and did not show an improvement across time; it was the increase of loneliness which changed the status of frailty [18]. Thus, we assumed the existence of a possible bidirectional association between social relationships and frailty which required further examination. Exploration of this relationship could help future research determine whether social relationship deficits and frailty precede each other or fall into a vicious cycle. This could help establish effective interventions for frail or socially unconnected older adults.

Cross-lagged model was used to explore the bidirectional association. Further, our study distinguished between within-person and between-person differences. The traditional cross-lagged panel model (CLPM) studies the longitudinal interrelationships between variables, and it assumes that the variables have no stable internal differences and only result in interpersonal effects, which is often unrealistic. Frailty and social relations are trait-like variables and show stable interpersonal variability over time [19, 20]. Studies have shown that conflating interpersonal and intrapersonal effects may lead to overestimation, underestimation, or even reversal of cross-lagged effects [21], and the conclusions drawn from CLPM at the interpersonal level cannot be inferred to the intrapersonal level [22]. For example, older adults with poor social relationships reporting frailty symptoms does not necessarily mean that improvement of social relationship will make them less frail [23]. The random intercept cross-lagged panel model (RI-CLPM) decomposes within-person and between-person effects in longitudinal associations between variables by including a random intercept to explain time-invariant individual differences, with less error than the traditional CLPM for within-person effects [21].

Against this background, we aimed to elucidate the longitudinal bidirectional relationship between frailty and social relationships in older adults using the RI-CLPM, taking into account the between-person effects.

## Materials and methods

### Design

This longitudinal study, conducted from 2011 to 2017, is a part of a project which started in 1991 in a suburban area of central Japan. This project aimed to identify the factors contributing to health, longevity, and well-being of the local residents. Data were collected using a self-administrated questionnaire. Initially, questionnaires were distributed to all residents and collected after 2 weeks. The survey was conducted every three years.

### Participants

**Fig. 1:**
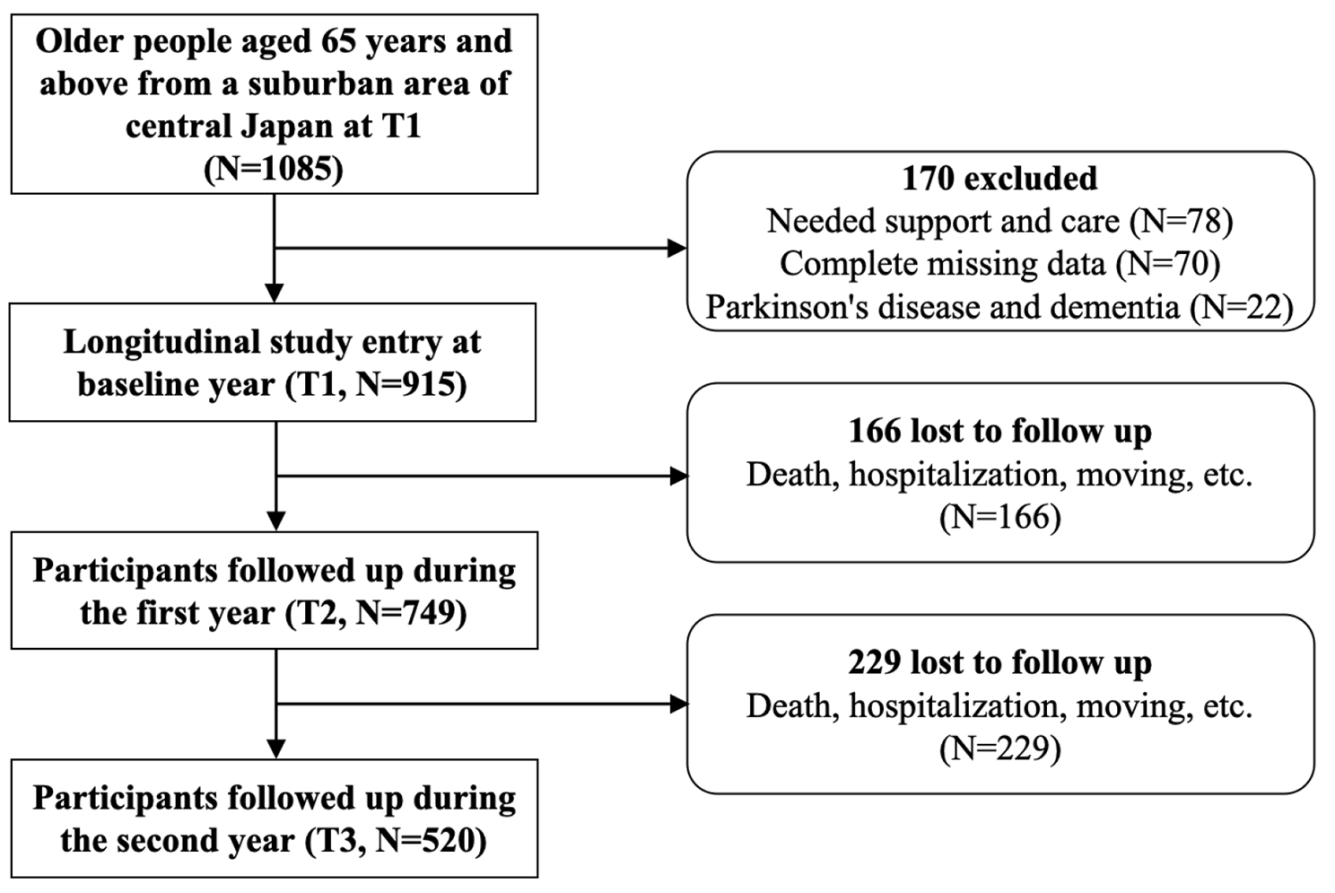
Flow chart of the participants in the present study

In the present study, older people aged 65 years and above were selected for the baseline year. Applying a longitudinal methodology, data from three waves were collected in 2011 (T1), 2014 (T2), and 2017 (T3). Initially, we selected 1085 participants aged ≥ 65 years at the baseline year; after eliminating 78 people needed support and care, 70 participants with complete missing data on frailty and social relationships and 22 adults with Parkinson’s disease and dementia, we included 915 participants and collected data regarding their social relationships and frailty in 2014 and 2017. Of this number, 166 participants were unable to complete follow-up in 2014 and 229 participants in 2017 due to death, hospitalization, moving, etc. Finally, 520 participants were included in this study (Fig. 1).

### Measures

#### Frailty

Frailty indicators were extracted from the Kihon Checklist (KCL), a popular frailty assessment tool [23]. The scale has been widely used in studies of frailty in the Japanese older adults and has shown sufficient reliability and validity [24, 25]. The lifestyle domain with 20 items was utilized which included activities of daily life, physical strength, nutritional status, oral function, the condition of being housebound, and cognitive function to predict frailty. Items were scored as either 0 for “good” or 1 for “poor”. The total score ranged from 0 to 20. A higher score indicated more symptoms of frailty.

#### Social relationships

The Index of Social Interaction (ISI) was used to evaluate social relationships, which measures various aspects of social relationships in daily settings [26]. There are 18 items in this scale, classified into five domains. The independence domain explores the motivation to live and maintain a healthy and active life. The social curiosity domain includes habits of reading, using new equipment, having hobbies, and a feeling of importance in society. The interaction domain assesses how well family and non-family people communicate. The domain of participation in society assesses involvement in social and neighborhood groups, as well as assuming an active social role. Finally, the feeling of safety domain explores if participants have someone offering counselling and providing support during emergencies. For the items, positive answers (always, often, sometimes) and negative answers (rare) were given 1 and 0 points, respectively. The total score ranged from 0 to 18. The higher score the participants got, the higher level of social relationships they had.

Frailty and social relationships showed appropriate internal consistency at three time points (frailty: α = 0.83, α = 0.88, α = 0.90; social relationships: α = 0.78, α = 0.83, α = 0.84).

#### Covariates

Based on previous studies, age, gender, exercise, alcohol consumption/smoking and chronic diseases were considered as covariates at baseline [27, 28]. Age and gender were considered as continuous and categorical variables, respectively. Regarding exercise, the respondents were asked “Do you exercise?” Responses of “always”, “frequently”, and “sometimes” were coded as “performing exercise” whereas responses of “no” were coded as “not exercising”. Regarding alcohol consumption, respondents were asked “Do you drink alcohol?” Responses of “always”, “everyday”, and “sometimes” were coded as “consumes alcohol” whereas responses of “hardly ever” and “do not consume alcohol” were coded as “non-consumers of alcohol”. For smoking, the respondents were asked “are you an active smoker?” Responses of “everyday” and “sometimes” were coded as “actively smoking”, responses of “I previously smoked but have now stopped” was coded as “former smoker”, whereas “I do not smoke” was coded as “non-smoking behavior”. Chronic diseases was treated as a categorical variable. Participants were classified as having at least one chronic disease or none based on the presence of the following diseases: hypertension, stroke, heart disease, diabetes, hyperlipidemia, lung disease, stomach/liver/gallbladder disorders, kidney disorders, musculoskeletal disorders, cancer, immune disease, depression, and eye and ear disorders.

### Statistical analysis

Descriptive statistics and bivariate correlations among all variables were reported using SPSS. RI-CLPM was used to explore the bidirectional relation between frailty and social relationships [20]. First, we evaluated intraclass correlation coefficients (ICC) using SPSS for frailty and social relationships to understand how much of variance was explained by the differences of participants. Second, measurement invariance was used to check the equivalence of these constructs across time to ensure that effects were attributed to real changes in variables. A three-step procedure was used for measurement invariance, including configural, metric, and scalar invariances [29]. Finally, the RI-CLPM was fit to identify bidirectional and time-ordered relations between frailty and social relationships. The covariates were controlled at the level of the random intercept. The comparative fit indices (CFI), Tucker-Lewis index (TLI), root mean square error of approximation (RMSEA), and standardized root mean square residual (SRMR) were used to estimate model fit [30, 31]. Acceptable model fit (CFI > 0.90, SRMR/RMSEA < 0.10) and good model fit (CFI/TLI > 0.95, SRMR/RMSEA < 0.08) were defined using standard benchmark values [32]. The results were presented as standardized coefficients. All analyses were performed using SPSS version 27 (Armonk, NY, USA) and Mplus version 8.6 (Muthén and Muthén, Los Angeles, CA, USA). The dataset had missing values due to the longitudinal nature. Full information maximum likelihood (FIML) was used to process missing data. FIML is a maximum likelihood method for handling missing data in a single step and is extensively used in structural equation models [33].

## Results

### Descriptive statistics

Table 1 shows the descriptive statistics for the demographic characteristics of participants, age at baseline, and frailty and social relationships scores. The mean and standard deviations of age at baseline were 73.02 ± 6.38 years. Over half of the participants were women, not living alone, had high economic status, and at least one chronic disease. The mean frailty scores were 2.37 ± 2.67 in 2011, 3.69 ± 3.33 in 2014, and 5.28 ± 3.88 in 2017. The average scores of social relationships were 16.42 ± 1.51, 16.02 ± 2.05, and 16.26 ± 1.83 in 2011, 2014, and 2017, respectively. Table 2 shows the correlation matrix for frailty and ISI. The bivariate correlations among frailty and ISI were in the expected direction. At each measurement wave, frailty was negatively correlated with ISI. The ICC for frailty was 0.64, indicating that 64% of the variance was due to between-person differences and 36% due to within-person fluctuations. For social relationships, the ICC was 0.55, indicating that 55% of the variation was due to the differences among older adults, and 45% due to intrapersonal fluctuations.

**Table 1:** Baseline characteristics of the study sample (*n* = 520)

Variables	Category	n (%) or Mean ± SD
Baseline age		73.02 ± 6.38
Gender	Men	225 (43.3)
	Women	295 (56.7)
Exercise	Yes	298 (57.3)
	No	172 (33.1)
	Missing	50 (9.6)
Alcohol consumption	Have	100 (19.2)
	None	393 (75.6)
	Missing	27 (5.2)
Smoking	Smoker	53 (10.2)
	Former smoker	117 (22.5)
	Non-smoker	307 (59.0)
	Missing	43 (8.3)
Chronic disease	Have	368 (70.8)
	None	152 (29.2)
Frailty T1		2.37 ± 2.67
Frailty T2		3.69 ± 3.33
Frailty T3		5.28 ± 3.88
Social relationships T1		16.42 ± 1.51
Social relationships T2		16.02 ± 2.05
Social relationships T3		16.26 ± 1.83

**Table 2:** Bivariate correlations (*n* = 520)

Measures	Frailty T1	Frailty T2	Frailty T3	ISI T1	ISI T2	ISI T3
**Frailty T1**	-					
**Frailty T2**	0.74**	-				
**Frailty T3**	0.67**	0.73**	-			
**ISI T1**	− 0.49**	− 0.44**	− 0.41**	-		
**ISI T2**	− 0.56**	− 0.57**	− 0.54**	0.71**	-	
**ISI T3**	− 0.49**	− 0.53**	− 0.58**	0.58**	0.74**	-

P* < 0.05, P** < 0.01; ISI, index of social interaction

### Measurement invariance

To compare factors longitudinally, we investigated measurement invariance in longitudinal measurement models of frailty and social relationships, for which the factor loadings must be time-invariant, at least with metric invariance across time [34, 35]. According to previous studies [36, 37], metric invariance can be established when compared with the configuration model, ΔCFI < 0.010, ΔRMSEA < 0.015, and ΔSRMR < 0.030, and scalar invariance, when, ΔCFI < 0.010, ΔRMSEA < 0.015, and ΔSRMR < 0.010. As shown in Table 3, both constructs demonstrated acceptable model fit and achieved metric invariance. Additionally, χ^2 difference tests did not yield significant result over time, indicating that frailty and social relationships can be compared across three time points.

**Table 3:** Longitudinal Measurement Invariance

Model	χ2	Δχ2	df	Δdf	CFI	ΔCFI	RMSEA	ΔRMSEA	SRMR	ΔSRMR
**Frailty**										
M1: Configural invariance	404.794	-	14	-	0.957	-	0.055	-	0.035	-
M2: Metric invariance	420.636	15.942	24	10	0.947	-0.010	0.059	0.004	0.044	0.009
M3: Scalar invariance	481.773	61.137*	41	17	0.809	-0.138	0.105	0.046	0.145	0.101
**ISI**										
M1: Configural invariance	320.309	-	72	-	0.970	-	0.061	-	0.058	-
M2: Metric invariance	329.913	9.604	80	8	0.961	-0.009	0.069	0.008	0.079	0.021
M3: Scalar invariance	357.391	27.478*	94	14	0.891	-0.070	0.106	0.037	0.161	0.082

M = model; CFI = comparative fit index; df = degrees of freedom; RMSEA = root mean square error of approximation; SRMR = standardized root mean square residual. Δ means the difference between two models. P* < 0.05

### RI-CLPM

**Fig. 2:**
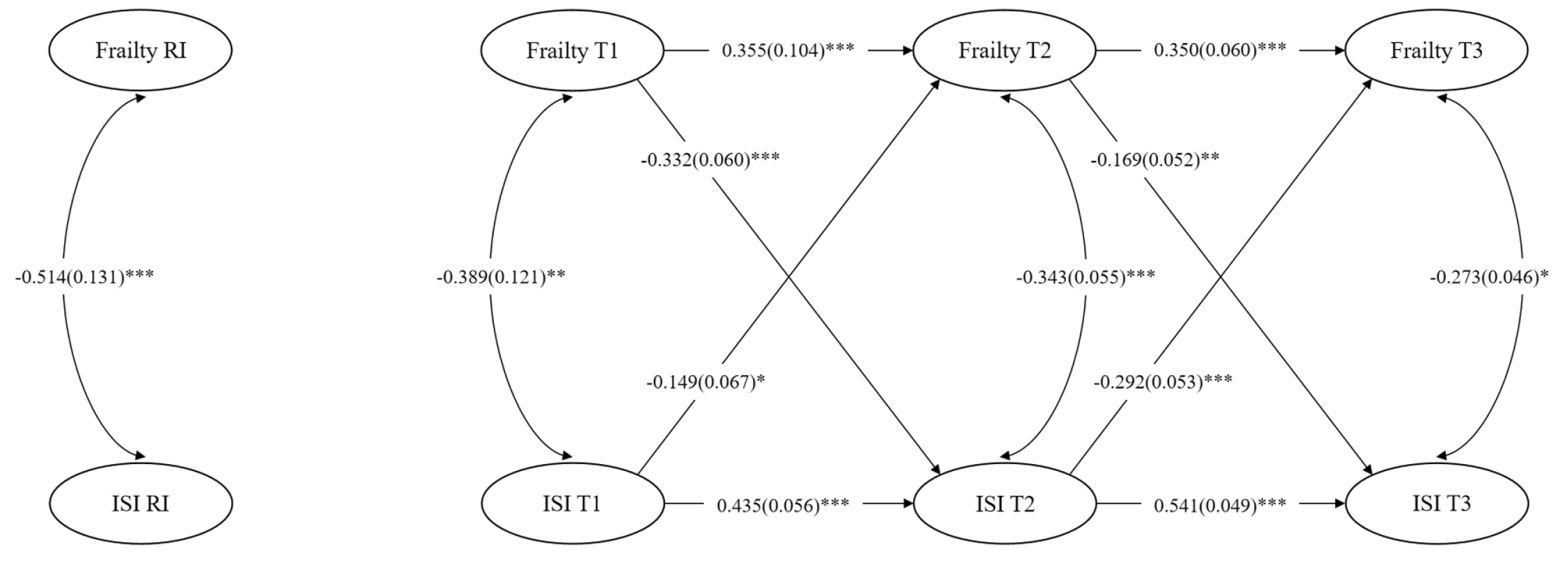
Random-intercept cross-lagged panel model results. *P < 0.05; **P < 0.01; ***P < 0.001. RI means random intercepts. Completely standardized parameter estimates with standard errors are reported in this model and controlled for all covariates. CFI = 0.962, TLI = 0.922, RMSEA = 0.078 (95% CI, 0.064–0.095), SRMR = 0.066, χ^2^ = 106.991, df = 25

The RI-CLPM model incorporating all covariates is depicted in Fig. 2 which indicates an acceptable model fit, χ2 = 106.991, df = 25, CFI = 0.962, TLI = 0.922, RMSEA = 0.078, SRMR = 0.066. The association between frailty and social relationships was divided into interpersonal and intrapersonal effects. At the interpersonal level, there was a strong negative association between frailty and social relationships (β = -0.514). This indicated that participants having more symptoms of frailty across the measurement waves showed lower level of social relationships and vice versa. At the individual level, social relationships and frailty had a negative cross-sectional association separately at T1 (β = -0.389, *p* < 0.01), T2 (β = -0.343, *p* < 0.001) and T3 (β = -0.273, *p* < 0.05). This meant that the intrapersonal changes of frailty and social relationships were correlated. The autoregressive path of frailty and social relationships were significant, indicating the severity of frailty and social relationships were carried over within-individuals to the next measurement wave. Intrapersonal cross-lagged paths from social relationships to frailty were significant at T1-T2 (β = -0.149, *p* < 0.05) and T2-T3 (β = -0.292, *p* < 0.001). In the opposite direction, the paths from frailty to social relationships were also significant at T1-T2 (β = -0.332, *p* < 0.001) and T2-T3 (β = -0.169, *p* < 0.01).

## Discussion

We explored the bidirectional association between frailty and social relationships in older people through RI-CLPM, while distinguishing interpersonal and intrapersonal effects. Our results showed a significant negative correlation at within- and between-person levels. Additionally, each measurement wave revealed consistent cross-sectional correlations. To our knowledge, this is one of the first studies to examine the within-person temporal dynamics of the relation between frailty and social relationships in older adults while controlling for between-person effects. Therefore, this study will provide the basis for future intervention research.

Regarding autoregressive influences, past social relationships always have a positive impact on future social relationships. Past research has shown that active social relationships can create positive feedback, making older adults more willing to participate in social activities and having a positive effect on the development of future social relationships [38]. Likewise, the effect of frailty symptoms on their own autoregression was consistent. As previous studies have shown, frailty accumulated with the increase of chronic diseases and the deterioration of body functions, and the accumulation rate accelerated with age [39]. The ability of older people who were already in a frail state to resist external disturbances further deteriorated, which in turn would aggravate the frail symptoms [40]. This result emphasizes the importance of early intervention.

In the present study, between-person relationship revealed a robust trait effect indicating a negative association between social relationships and frailty across all three datasets. Individuals characterized by diminished social relationships exhibited a higher propensity for experiencing frailty symptoms compared to their counterparts. Consequently, these between-person findings delineate populations warranting targeted interventions. Our results offer additional elucidation on the interplay between social relationships and frailty symptoms among older adults, highlighting the heightened vulnerability of individuals with attenuated social networks to increased frailty manifestations.

After controlling for these trait effects, the within-person autoregressive path showed a vicious cycle of frailty and social relationships. Social relationships always predicted the subsequent variations of frailty. Although there were methodological differences in the assessment, similar results could be found in previous studies. A longitudinal study in the United Kingdom suggests that an increased risk of frailty is associated with reduced social relationships. Individuals with poor social relationships may experience prevalence of cardiovascular disease and exhibit worse health behaviors [3]. Because of infrequent contact with others, people with lower social relationships have reduced need for health, which may contribute to the risk of frailty. Longitudinal studies linking social relationships with walking speed, activities of daily living [41], and mobility and upper extremity strength [42] suggested that weakened social relationships might increase the risk of sarcopenia, a major factor in frailty [43], and some measures of frailty, including upper extremity strength and walking ability, were direct measures of sarcopenia. Moreover, one of the etiologies of sarcopenia is lack of physical activity [44], which could be one possible link between lack of social relationships and development of frailty. People with poor social relationships tend to be less physically active [45] which leads to an increased risk of frailty [46]. Therefore, it is advisable to include promotion of social relationships in the intervention kit for preventing or decreasing frailty.

In the opposite direction, the predictive effect of frailty on social relationships was also reflected in all measured waves, which was consistent with a few previous studies. A longitudinal study in Singapore showed association between increased social engagement and decreased frailty [47]. Symptoms of frailty increase loneliness in older people and make them inactive to participate in social activities, thus affecting social relationships. Another longitudinal study of older adults demonstrated that frailty led to reduced social activity and contact with neighbors [17]. The lack of adequate physical activity in physically-limited older adults restricts mobility in the living space, which in turn limits social relationships. Although there is not enough research on the effects of frailty on social relationships, there are many studies that report the predictive effects of subdomains of frailty on social relationships. For example, functional decline [48], poorer cognitive function [49], poor oral health [50], and cognitive decline [51] were shown to be predictors of worse social relationships. As levels of these subdomains decrease, older adults become frailer [52, 53].

In each measurement wave, frailty and social relationships had a significant cross-sectional association. Thus, better social relationships may be associated with milder frailty symptoms and vice versa. This is consistent with the findings of Lestari et al., showing that frail older adults preferred less-active types of social relationships [4], and Tsutsumimoto et al., showing that frailty and social relationships in community-dwelling older adults were negatively correlated [54]. In a short-term Austrian study, the prevalence of frailty significantly reduced within 12 weeks after a social support intervention [6], suggesting the relatively stronger association between frailty and social relationships in the short term than in the long term. It is worth mentioning that our study also found that the lagging effect of frailty on social relationships became progressively weaker, probably due to the fact that long periods of frailty allowed older adults to adapt to physical changes and thus had the ability to maintain certain social relationships [55]. This may be related to the positive attitude of older adults towards life. In contrast, the lagged effect of social relationships on frailty became stronger. Previous studies suggested that there may be some potential mediating variables between social relationships and frailty, for example, higher levels of social relationships may be effective in reducing the risk of loneliness and depression, and thus further reducing the risk of frailty [56]. These findings may provide directions for future research.

Due to the reversible state of frailty in the older adults, the results of our study have practical implications. The importance of improving social relationships should be considered at the same time when providing care and interventions for frail older adults. Helping them to establish positive social connections such as family and friends, social networks, and some volunteer activities can be effective in improving frailty [57]. Moreover, intervention programs should be gradual, from weak to strong, because frail older adults tend to have less social participation. Some social activities, such as volunteering or physical exercise in salons often requires a higher level of functioning [58].

This study’s key strength is the utilization of RI-CLPM to examine the longitudinal bidirectional relation between frailty and social relationships and detection of within-person relations while controlling the between-person effect. Another advantage is that this study confirmed the vicious circle between frailty and social relationships, so it may be more effective to consider the importance of social relationships when developing intervention programs for frail older adults.

This study has some limitations. First, the relatively small size of the study may affect the stability of the results. Second, the study’s coverage was less extensive as it did not include a psychological dimension to measure frailty. Third, this study can only generalize the association between overall scores of frailty and social relationships, and cannot derive the specific effects between the sub-domains. Therefore, it is recommended that future studies subdivide frailty or social relationships to explore more in-depth mechanisms. Finally, the long span between time points and the small total number of waves in this study did not detect a more stable lag effect, which could lead to serendipity, and therefore future studies are recommended to perform more number of measurements to enhance the stability of this type of study.

## Conclusion

Our findings suggest that frailty and social relations in Japanese older adults are strongly correlated between people. Within people, changes in frailty and social relationships are consistently correlated. It further suggests that there is a vicious cycle between frailty and social relationships, which means the deterioration of social relationships in older adults also increases the risk of becoming more frail and vice versa. Our results emphasized that providing the earliest possible intervention for frail older populations while maintaining certain social relationships was necessary both to improve the frailty itself and to avoid a vicious cycle of further frailty in the future.

## Data Availability

The data that support the findings of this study are available from the local government but restrictions apply to the availability of these data, which were used under license for the current study, and so are not publicly available. Data are however available from the authors upon reasonable request and with permission of the local government.
